# Integrated Glycome Strategy for Characterization of Aberrant LacNAc Contained N-Glycans Associated With Gastric Carcinoma

**DOI:** 10.3389/fonc.2019.00636

**Published:** 2019-07-10

**Authors:** Hanjie Yu, Xiaojie Li, Mengting Chen, Fan Zhang, Xiawei Liu, Jingmin Yu, Yaogang Zhong, Jian Shu, Wentian Chen, Haoqi Du, Kun Zhang, Chen Zhang, Jing Zhang, Hailong Xie, Zheng Li

**Affiliations:** ^1^Laboratory for Functional Glycomics, College of Life Sciences, Northwest University, Xi'an, China; ^2^Department of Pathology, 1st People's Hospital of Chenzhou, Chenzhou, China; ^3^Institute of Cancer Research, University of South China, Hengyang, China

**Keywords:** gastric carcinoma, glycosylation, lectin microarray, MALDI-TOF/TOF-MS, LacNAc

## Abstract

Aberrant glycosylation is not only a feature of malignant cell transformation, but also plays an important role in metastasis. In the present study, an integrated strategy combining the lectin microarrays and lectin cytochemistry was employed to investigate and verify the altered glycopatterns in gastric cancer (GC) cell lines as well as resected tumor specimens from matched tissue sets of 46 GC patients. Subsequently, lectin-mediated affinity capture glycoproteins, and MALDI-TOF/TOF-MS were employed to further acquire precise structural information of the altered glycans. According to the results, the glycopatterns recognized by 10 (e.g., ACA, MAL-I, and ConA) and 3 lectins (PNA, MAL-I, and VVA) showed significantly variations in GC cells and tissue compared to their corresponding controls, respectively. Notably, the relative abundance of Galβ-1,4GlcNAc (LacNAc) recognized by MAL-I exhibited a significant increase in GC cells (*p* < 0.001) and tissue from patients at stage II and III (*p* < 0.05), and a significant increase in lymph node positive tumor cases, compared with lymph node negative tumor cases (*p* < 0.05). More LacNAc contained N-glycans were characterized in tumor sample with advanced stage compared to corresponding control. Moreover, there were 10 neo-LacNAc-contained N-glycans (e.g., *m/z* 1625.605, 1803.652, and 1914.671) only presented in GC tissue with advanced stage. Among these, six N-glycans were modified with sialic acid or fucose based on LacNAc to form sialylated N-glycans or lewis antigens, respectively. Our results revealed that the aberrant expression of LacNAc is a characteristic of GC, and LacNAc may serve as a scaffold to be further modified with sialic acid or fucose. Our findings provided useful information for us to understand the development of GC.

## Introduction

Gastric carcinoma (GC) is the third most common cause of cancer mortality in the world (1). In 2015, it was estimated that 24,590 new cases of GC would be diagnosed with GC and 10,720 individuals would die from GC in the United States (2). Although the GC death rate has declined in the mass, the 5-year survival rate for surgically treated GC patients is still low for patients with advanced-stage of GC (3–5).

Glycosylation is one of the most common and complex forms of post-translational modification. Carbohydrate moieties are indispensable for the correctly folded and mature conformation of proteins, and it also functions in intercellular contact, cell recognition, and signal transduction (6–8). Alterations of glycosylation are common features of some diseases such as cancer (9, 10). For instance, the level of sialylation is known to be elevated in various cancers (11, 12). The sialyl-Lewis X (SLe^x^) epitope is a well-known tumor-associated carbohydrate antigen that is significantly increased on triantennary glycans and is accompanied by increased levels of core fucosylated agalactosyl biantennary glycans, which is present on IgG isolated from GC patients. Increased levels of these glycans are associated with increasing disease pathogenesis and liver metastasis, and the increased expression of SLe^x^ is a result of pro-inflammatory cytokine signaling during carcinogenesis (13, 14). GC cell lines have been widely used as *in vitro* model to study carcinogenesis mechanism and drug discovery. Zhao et al. reported that the up-regulation of GDP-fucose transporter and Fut8 could inhibit proliferation, but had no significant influence on migration of BGC-823 and SGC-7901 cells (15). The N-glycosylation of GC cell lines with various grades of differentiation (AGS, SGC-7901, and NCI-N87) were characterized and compared by ultra-high performance liquid chromatography (UHPLC), as a result, different levels of N-glycosylation were exhibited among GC cells (16).

N-acetyllactosamine (LacNAc) is a disaccharide that is synthesized from linking GlcNAc and Gal by a β1-4 linkage, and the terminal galactose of LacNAc could be modified with a GlcNAc moiety to form two LacNAc moieties. This reaction may continue to form poly-N-acetyllactosamine (poly-LacNAc). Abnormal expression of LacNAc and its polymers on both *O*- and *N*-glycans are associated with cancers such as thyroid papillary carcinomas, melanomas, and hepatocarcinomas may promote metastasis (17–19). LacNAc can serve as a substrate for further modifications, such as sialylation and fucosylation, and most α2-6-sialic acid is found linked to N-acetyllactosamine moieties (11, 20).

In the present study, an integrated strategy combining the lectin microarrays and lectin cytochemistry was employed to investigate and verify the altered glycopatterns in gastric cancer (GC) cell lines and resected tumor specimens from matched tissue sets of 46 GC patients with the different clinical stages. Subsequently, lectin-mediated affinity capture glycoproteins and MALDI-TOF/TOF-MS were also employed to acquire precise structural information of the altered glycans. This study elucidated the aberrant glycosylation, especially the associations between the increased LacNAc and the development of GC, furthermore, characteristics of the aberrant glycosylation may help us to understand the development of GC.

## Materials and Methods

### Ethics Statements

The collection of human tissue was carried out in accordance with the approved guidelines, approved by the Human Ethics Committee of all participating units (Northwest University, 1st People's Hospital of Chenzhou and Institute of Cancer Research, University of South China, Hunan). Written informed consent was received from participants. This study was conducted in accordance with the ethical guidelines of the Declaration of Helsinki.

### Cell Lines and Tissue Specimens

The GC cell lines (SGC-7901, MGC-803, and BGC-823) and human normal gastric epithelial cell line (GES-1) were maintained in DMEM -high glucose cell culture media (HyClone, Waltham, MA, USA) supplemented with 10% fetal bovine serum (GIBCO, Grand Island, NY, USA) and 100 U/mL of penicillin and streptomycin The cells were kept at 37°C with 5% CO_2_ condition. The tissue specimens were obtained from primary tumors, which were resected before chemotherapy or radiotherapy from patients at the Department of Pathology in the First People's Hospital of Chenzhou, China. Patients were staged according to the 2010 UICC/AJCC system. After biopsy, 46 pairs of tumor and matched adjacent non-tumor tissue from the same patients were collected. The detailed clinical information was summarized in Table 1.

**Table 1:** The clinical information of gastric cancer patients.

	**Gastric adenocarcinoma (*n* = 46)**
**AGE (Y)**	
Range	26–73
mean±SD	55.5 ± 10.0
**GENDER**	
Male/Female	34/12
**TNM STAGE**	
Stage I	8
Stage II	9
Stage III	29
**DEPTH OF TUMOR INVASION(T)**	
T1	6
T2	10
T3	9
T4	21
**LYMPH NODE METASTASIS(N)**	
N0	10
N1	7
N2	14
N3	15
**BORRMANN CLASSIFICATION**	
Type I	3
Type II	35
Type III	5
Type IV	3

### Extraction of Cell/Tissue Proteins

After grown to 80–90% confluence, the median was removed and the cells were washed with 10 mM of PBS (0.1 M phosphate buffer containing 0.15 M NaCl, pH 7.4) for twice. Then, 1 mL of M-PER Mammalian Protein Extraction Reagent (Thermo, Scientific; Herts, UK) with 1% (*v/v*) of protease inhibitor (Sigma-Aldrich) were added into T25 flask and incubated for 15 min on ice. Subsequently, solution was transferred into a new tube, and centrifuged at 10,000 × g for 15 min, the supernatant was immediately transferred to a new tube and stored at −80°C. The tissue specimens were washed twice by 10 mM of PBS, then homogenized by 1 ml of T-PER Tissue Protein Extraction Reagent (Thermo Scientific; Herts, UK) containing 1% (*v/v*) of Protease Inhibitor Cocktail, and incubated it for 30 min on ice. After that, centrifugation at 10,000 × g, 4°C for 10 min was applied to obtain the supernatants, the supernatants were then immediately transferred into new tubes and stored at −80°C. The protein concentration was determined by BCA assay (Beyotime Biotechnology, China).

### Lectin Microarray and Data Analysis

The manufacture of lectin microarray and data acquisition were performed as described previously (21–23). Briefly, lectins were purchased from Vector Laboratories (Burlingame, CA) and Sigma-Aldrich (St. Louis, MO) and dissolved into the manufacturer's recommended solution at a concentration of 1 mg/mL, which contained 1 mM of the appropriate monosaccharide. Then, lectins were spotted onto homemade epoxysilane-coated slides, and each lectin was printed in triplicate. Microarrays were blocked using the blocking reagent (2% (*w/v*) BSA in PBST, 10 mM of PBS buffer contained 0.02% (*v/v*) Tween-20) for 1 h. the slides were washed for three times and centrifuged. The extracted proteins from cells or tissue were labeled by Cy3 fluorescent dye (GE Healthcare, Biosciences, Piscataway, NJ, USA) and purified using a Sephadex-G25 column (GE Healthcare). Subsequently, 4 μg of labeled protein was mixed with 120 μL of lectin microarray incubation buffer and applied to the lectin microarrays then incubated in the chamber at 37°C for 3 h. After washing for three times, the slides were centrifuged to dry and scanned by using a GenePix 4000B confocal scanner (AXON, Instruments, Inc.). The fluorescence intensities were extracted by GenePix 6.0 software (Axon).

To eliminate the influence of non-specific adsorption, the signal values less than average background + standard deviations (SD) were excluded from each data point, and global normalization was used to eliminate fluoresce bias. After normalized, the processed data from the parallel data sets were compared with each other based upon the fold-changes under the following criteria: fold changes ≥ 1.5 or ≤ 0.67 indicated an up-regulation or a down-regulation, respectively. Differences of each lectin between tumor samples and control groups were tested by Student's *t*-test or paired *t*-test. The normalized data was further analyzed by Expander 6.0 software to perform a hierarchical clustering analysis (http://acgt.cs.tau.ac.il/expander/).

### Selective Isolation of Glycoprotein Fractions From Tissue by MAL-I-Magnetic Particle Conjugates

The LacNAc-contained glycoproteins were isolated from tissue using MAL-I-magnetic particle conjugates as previously described (24, 25). Firstly, equal quantities of all tumor cases with same stages were pooled, and their corresponding para-carcinoma tissue proteins were also pooled at equal quantities. Two milligram of the pooled tissue proteins from tumor as well as controls were diluted to 600 μL with the binding buffer (0.1 M Tris-HCl, 0.15 M NaCl, 1 mM CaCl_2_, 1 mM MgCl_2_, and 1 mM MnCl_2_, pH 7.4) supplemented with 6 μL of proteinase inhibitor cocktail (Sigma-Aldrich). Subsequently, the conjugates were washed for three times with binding buffer, followed by the incubation with the diluted tissue proteins for 3 h with gentle shaking at RT. After that, the conjugates were washed three times with the washing buffer (binding buffer supplemented with 0.1% (*v/v*) Tween-20) to remove unbounded proteins. Then, the glycoproteins which bound to the conjugates were eluted with 300 μL of competitive elution (200 mM Lactose in binding buffer) for 1 h with shaking at RT. The isolated protein concentration was determined by BCA assay (Beyotime).

### Release and Purification of N-Glycans

The N-glycans were released by PNGase F glycosidase (New England Biolabs, Beverly, MA) according to previous protocols (25, 26). Briefly, 200 μg of isolated glycoproteins were concentrated and desalted by adding to a size-exclusion spin ultrafiltration (Amicon Ultra-0.5 mL 10,000 MW cut off, Millipore). Then, the obtained glycoproteins were denatured with 8M urea (Sigma-aldrich), 10 mM DTT, and 10 mM IAM (Sigma-Aldrich). Followed by the exchange of buffer into 40 mM NH_4_HCO_3_ via using 10 K centrifugal ultrafiltration. After that, 5 μL of PNGase F (NEB) were added into ultrafiltration and incubated over night with shaking at 37°C. The reaction was stopped by incubating the mixture at 80°C for 5 min. After centrifuge, the released N-glycans were collected and purified with HyperSep Hypercarb SPE cartridges (25 mg, 1 mL; Thermo Scientific) according to the manufacturer recommendation. The N-glycans were eluted by 0.5 mL of elution solution (50% (*v/v*) acetonitrile with 0.1% (*v/v*) TFA). The purified N-glycans were collected and lyophilized.

### Characterization of N -Glycans Using MALDI-TOF/TOF-MS

The MAL-I-enriched N-linked glycans were characterized using MALDI-TOF/TOF-MS (UltrafleXtreme, Bruker Daltonics, Germany) as previously described (25, 26). Briefly, the glycans were resuspended in 10 μL of methanol, then glycan samples and DHB were spotted on a MTP AnchorChip, respectively to recrystallize the glycans. Ionization was performed in MS and MS/MS by irradiation of a nitrogen laser (337 nm) operating at 1 kHz. Data were acquired at a maximum accelerating potential of 25 kV in the positive and reflectron modes. Mass calibration was done using the peptide calibration standards 250 calibration points from Bruker Daltonics. A total of 1,500 laser shots per pixel (200 laser shots per position of a random walk within each pixel) were collected and the data were acquired using the flexAnalysis software (Bruker Daltonics, version 3.3). Representative MS spectra of N-glycans with signal-to-noise ratios > 6 were annotated using the GlycoWorkbench software. By combining information from glycan structures recognized by a lectin and received from fragmentation of [M+H]+ and [M+Na]+ ions of the glycans, a complete structural characterization of oligosaccharides were achieved. The relative intensity (RI) of each glycan peak was calculated by dividing the intensity of a given type of glycan by the total glycans intensity (27–39).

### Lectin Cytochemistry

Lectin cytochemistry was performed as described previously (21, 30). First of all, 100 μg of *Maackia amurensis lectin-I* (MAL-I) was labeled with Cy5 dye (GE healthcare) and then purified by a Sephadex-G25 column (GE healthcare). The cells (1 × 10^5^ cells) were inoculated into confocal culture dishes (JingAn Biotechnology Co., Ltd Shanghai China) and cultured in complete medium for 12 h. After that, the cells were washed three times with 10 mM of PBS (5 min each), and immobilized by incubating with 0.2% (*v/v*) Triton X-100 in 4% (*v/v*) paraformaldehyde for 30 min at room temperature (RT). Subsequently, the dishes were blocked by Carbo-Free Blocking Solution (Vector labs, Burlingame, CA) for 1 h prior to staining at RT. The cells were then incubated with blocking solution containing 100 μg/mL of Cy5 labeled MAL-I for 4 h with protection from light at RT. After washing for three times with 10 mM of PBS, cells were stained with 4′, 6-diamidino-2-phenylindole (DAPI, 1 μg/mL in 10 mM of PBS) (Roche; Basel, Swatzerland) for 10 min and then washed with 10 mM of PBS again. Finally, the cells were scanned by Laser Scanning Confocal Microscope FV 1000(Olympus, Tokyo, JPN) at the same exposure time and shown on the same scale.

## Results

### Comparsion of Glycopatterns Between GC Cell Lines and GES-1

**Figure 1:**
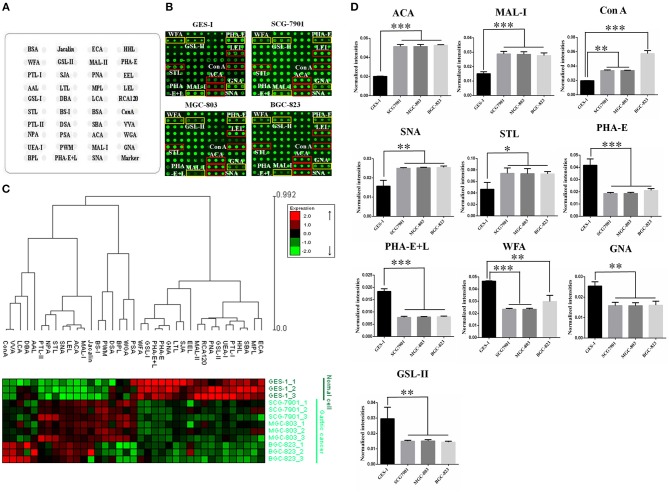
Evaluation of altered glycopatterns in GC cell lines using lectin microarrays. **(A)** The layout of lectin microarray. A total of 37 lectins were dissolved in the recommended buffer and spotted on lectin microarray. **(B)** Scanned images were obtained for the analysis of GES-1 and GC cell lines. The lectins with increased NFIs in GC cell lines are marked with red boxes, and those with decreased NFIs are marked with yellow boxes. **(C)** Heat map and hierarchical clustering analysis of the 37 lectins with three biological replicates. Glycan profiles of GC cell lines (SGC-7901, MGC-803, and BGC-823) and their normal reference (GES-1) were clustered (average linkage, correlation similarity). Samples are listed in rows and the lectins are listed in columns. The color and intensity of each square indicated expression levels relative to other data in the row. Red, high; green, low; black, medium. **(D)** The NFIs of 10 lectins were significantly altered in GC cells compared to the normal reference based on fold change and *t*-test (^*^p < 0.05, ^**^p < 0.01, and ^***^p < 0.001). The data are presented as the averaged NFI ± SD of three biological replicates.

To identify the abnormal glycopatterns associated with GC, lectin microarrays were utilized to compare the glycan profiles between GC cells/tissue and their corresponding controls. A lectin microarray format and the glycopatterns of Cy3-labeled proteins from GC cell lines and GES-1 bound to the lectin microarrays were shown in Figures 1A,B. The averaged normalized fluorescent intensities (NFIs) of each lectin from GC cell lines and GES-1 were summarized as mean values ± SD in Table S1. The generated data from three biological replicates were imported into EXPANDER software to perform a hierarchical clustering analysis furtherly (Figure 1C). The results of ratio analysis revealed that the NFIs of 10 lectins (e.g., ACA, MAL-I, and Con A) showed significantly altered between three GC cell lines and GES-1 (Table 2). Among the total, the T antigen, sialyl-T antigen binder ACA, the LacNAc binder MAL-I, high mannose type N-glycans binder Con A, Sia2-6Gal/GalNAc binder SNA and oligomers of GlcNAc binder STL showed significantly increased NFIs in all GC cell lines compared to GES-1 (all fold change>1.5, *p* < 0.01). Conversely, the bisecting GlcNAc and bi/tri -antennary N-glycans binders PHA-E and PHA-E+L, the GalNAcα/β1-3/6Gal binder WFA, Manα1-3Man binder GNA, and agalacto-type, tri- or tetra-antennary N-glycans binder GSL-II exhibited significantly decreased NFIs in GC cell lines compared to GES-1 (all fold change <0.6, *p* < 0.01) (Figure 1D).

**Table 2:** Altered glycopattern of cellular glycoproteins between normal and GC cells based on data of 10 Lectins giving significant differences.

**Lectin**	**Specificity**	**Fold change**		
		**SGC-7901/GES-1**	**MGC-803/GES-1**	**BGC-823/GES-1**
ACA	Galβ1-3GalNAcα-Ser/Thr (T antigen), sialyl-T(ST)	2.541^***^	2.572^***^	2.611^***^
MAL-I	Galβ-1, 4GlcNAc	1.946^***^	1.917^***^	1.884^***^
Con A	High-Mannose	1.737^**^	1.729^**^	2.943^***^
SNA	Sia2-6Gal/GalNAc	1.571^**^	1.579^**^	1.617^**^
STL	Trimers and tetramers of GlcNAc, core (GlcNAc) of N-glycan	1.532^*^	1.527^*^	1.572^*^
PHA-E+L	Bisecting GlcNAc, bi-antennary N-glycans, tri- and tetra-antennary complex-type N-glycan	0.431^***^	0.444^***^	0.453^***^
PHA-E	Bisecting GlcNAc, biantennary complex-type N-glycan	0.475^***^	0.478^***^	0.539^***^
WFA	Terminating in GalNAcα/β1-3/6Gal	0.508^***^	0.502^***^	0.647^**^
GNA	Manα1-3Man	0.588^**^	0.582^**^	0.585^**^
GSL-II	GlcNAc and agalactosylated tri/tetra antennary glycans	0.442^**^	0.441^**^	0.428^**^

* *p < 0.05*,

** p < 0.01, and

*** *p < 0.001*.

### Assessment of Altered Glycopatterns Associated With GC

**Figure 2:**
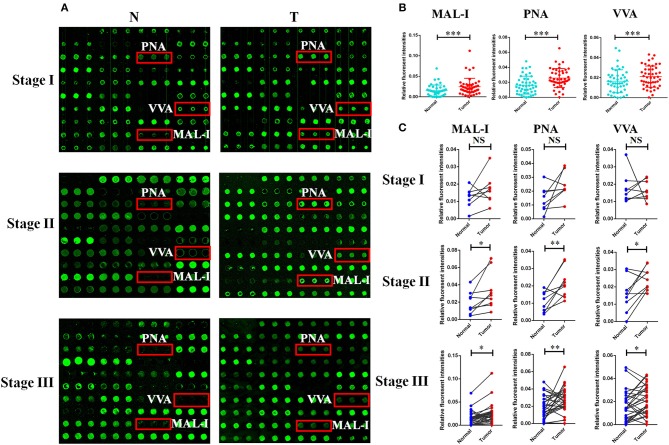
The different glycopatterns in tumor and their corresponding adjacent non-tumor tissue using a lectin microarray. **(A)** The profile of Cy3-labeled tissue proteins from tumor tissue (T) at different TNM stages and matched adjacent non-tumor tissue (N) bound to the lectin microarrays. The lectins (MAL-I, PNA, and VVA) which recognize the altered glycopatterns are marked in red boxes. **(B)** The NFIs of 3 lectins were significant higher in tumor specimens than in controls based on 46 paired of samples. **(C)** Comparison of the NFIs of 3 lectins between tumor and corresponding controls at different TNM stages. The NFIs of 3 lectins were significantly increased in tumor specimens compared to normal controls in advanced-stage of GC based on fold change and paired *t*-test (NS, not significantly different, ^*^p < 0.05, ^**^p < 0.01, and ^***^p < 0.001).

In order to investigate how the glycopatterns changed during the development of GC, the glycan profiles of tumor tissue at different stages and their corresponding controls were investigated as well. The glycopatterns of Cy3-labeled tissue proteins from tumor and their corresponding controls bound to the lectin microarrays are shown in Figure 2A. Overall, the NFIs of 3 lectins (MAL-I, PNA, and VVA) showed significantly increased in tumor tissue compared to the controls (*p* < 0.001) (Figure 2B). Specifically, the LacNAc binder MAL-I exhibited increased NFIs in 34 of 46 (73.91%) tumor tissue, T antigen binder PNA and Tn antigen binder VVA also exhibited increased NFIs in 27 of 46 (58.70%) and 24 of 46 (52.17%) tumor tissue compared with their corresponding adjacent tissue, respectively (Table 3). Moreover, the NFIs of MAL-I showed significant difference between lymph node positive tumor cases (Nx) and lymph node negative tumor cases (N0) (χ^2^ = 7.62, *p* < 0.05). To elucidate the altered glycopatterns in tumor progression, the tumors were categorized into clinical stages, glycan patterns of tumor, and controls were further compared based on the NFIs. The results revealed that there were 4/8 (50.00%) of the tumor cases at stage I (foldchange: 1.73 ± 1.05, *p* > 0.05); 6/9 (66.67%) tumor cases at stage II (foldchange: 2.05 ± 1.26, *p* < 0.05), and 24/29 (82.76%) tumor cases at stage III (foldchange: 6.92 ± 12.48, *p* < 0.05) showed significantly increased NFIs compared to their corresponding controls, which indicated that the relative abundance of LacNAc exhibited a coincidentally increased trend during the tumor progression. However, the T antigen binder PNA and Tn antigen VVA also showed a coincidentally increasing trend during stage I and stage II, but it did not show an increasing trend in the advanced stage (Figure 2C).

**Table 3:** Fold change of tissue glycopatterns from 46 paired tumor tissue and adjacent non-tumor tissue based upon ratio of the NFIs of 3 lectins.

**Lectin**	**Specificity**	**Frequency**	**Fold change**^a^		
			**Stage I**	**Stage II**	**Stage III**
MAL-I	Galβ-1,4GlcNAc	34/46	(1.73 ± 1.05)^ns^	(2.05 ± 1.26)^**^	(6.92 ± 12.48)^**^
PNA	Galβ1-3GalNAcα-Ser/Thr(T)	27/46	(4.51 ± 8.35)^ns^	(2.45 ± 1.44)^*^	(4.39 ± 7.01)^*^
VVA	GalNAc and GalNAcα-Ser/Thr(Tn)	24/46	(1.12 ± 0.47)^ns^	(3.83 ± 6.14)^*^	(3.05 ± 4.39)^*^

a *The foldchange was presented as Mean with SD*.

*ns, no significantly different (p > 0.05)*,

* *p < 0.05*;

** *p < 0.01*;

*** *p < 0.001*.

### Characterization of Altered N-Glycans in GC Tissue

**Figure 3:**
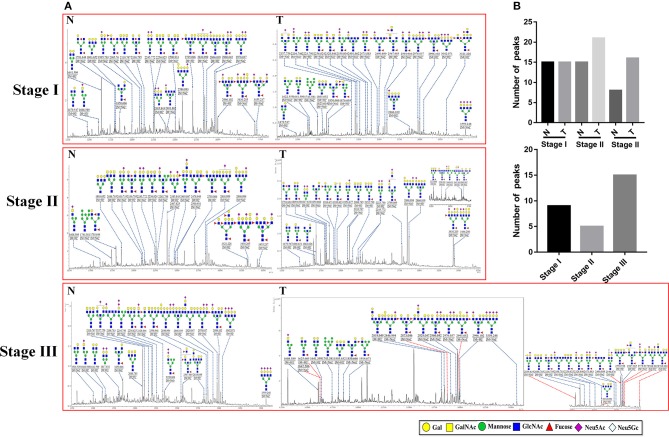
MALDI-TOF/TOF-MS spectra of MAL-I isolated N-glycan peaks from tumor and adjacent non-tumor tissue in different clinical stages. **(A)** The glycoproteins from tumor and adjacent non-tumor tissue which contained LacNAc were isolated by MAL-I-magnetic particle conjugates, and N-glycans were released and characterized by MALDI-TOF/TOF-MS. Detailed glycan structures were analyzed using the GlycoWorkbench software. Proposed structures and their *m/z* values were shown for each peak. Ten neo-LacNAc-contained N-glycan structures which only detected in tumor tissue at stage III were marked with red dotted line. **(B)** The number of peaks which corresponding to the LacNAc contained N-glycans that only presented in tumor or control samples in different stages (upper); and the number of peaks which corresponding to LacNAc contained N-glycans that presented in both tumor and control samples (lower).

To obtain detail structural information of LacNAc contained N-glycans, the glycoproteins from the tumor and adjacent non-tumor tissue were isolated using the MAL-I -magnetic particle conjugates, and N-glycans were released from the isolated glycoproteins by PNGase F, and characterized by MALDI-TOF/TOF-MS. The MALDI-TOF/TOF-MS peaks of tumor and adjacent non-tumor specimens were shown in Figure 3A. The detailed information of the annotated N-glycans were summarized in Table S2. There were 24 LacNAc-contained N-glycans to be identified in tumor and non-tumor tissue at stage I, 26 and 20 LacNAc-contained N-glycans to be identified in tumor and non-tumor tissue at stage II, and 31 and 23 LacNAc-contained N-glycans to be identified in tumor and non-tumor tissue at stage III, respectively. The common and unique structures were summarized in Figure 3B. Surprisingly, LacNAc contained N-glycans which characterized in tumor samples in the early stages (stage I and II) and adjacent tissue did not show significant change. However, more LacNAc contained N-glycans were characterized in tumor tissue with the advanced stage (stage III) compared to adjacent tissue. During the tumor development, there were 24 and 26 LacNAc contained N-glycans to be characterized in tumor samples at stage I and stage II, specifically, 31 LacNAc contained N-glycans were characterized in tumor sample at stage III. By contrast, LacNAc contained N-glycans which characterized in adjacent samples did not show an obvious change. The LacNAc-modified glycans at *m/z* 1479.547 [(Gal)_1_ (GlcNAc)_2_ + (Man)_3_ (GlcNAc)_2_] and 2223.790 [(NeuAc)_2_ (Gal)_2_ (GlcNAc)_2_ + (Man)_3_ (GlcNAc)_2_] were only detected in tumor samples at stage I and II, and the LacNAc-modified glycans at *m/z* 1914.671 [(Fuc)_2_ (Gal)_1_ (GlcNAc)_1_ (Man)_2_ + (Man)_3_ (GlcNAc)_2_] presented in tumor samples at stage II and III uniquely. Moreover, 10 neo-LacNAc-contained N-glycans (e.g., *m/z* 1625.605, 1803.652, and 1914.671) that were identified and annotated with proposed structures were unique in GC samples with advanced stage. Five of which (*m/z* 2034.634 [(NeuAc)_1_ (Gal)_1_ (Sulf-Gal)_1_ (GlcNAc)_2_ + (Man)_3_ (GlcNAc)_2_)], 2546.876 [(NeuGc)_2_ (NeuAc)_1_ (Gal)_2_ (GlcNAc)_2_ + (Man)_3_ (GlcNAc)_2_], 2578.915 [(Fuc)_2_ (NeuAc)_2_ (GalNAc)_1_ (Gal)_1_ (GlcNAc)_2_ + (Man)_3_ (GlcNAc)_2_], 2588.922 [(NeuAc)_2_ (Gal)_3_ (GlcNAc)_3_ + (Man)_3_ (GlcNAc)_2_], and 2993.052 [(Fuc)_1_ (NeuAc)_1_ (Gal)_5_ (GlcNAc)_4_ + (Man)_3_ (GlcNAc)_2_] modified with sialic acid and two of which [*m/z* 2578.915 and 2873.046 ((Fuc)_3_ (Gal)_3_ (GlcNAc)_5_ + (Man)_3_ (GlcNAc)_2_)] modified with fucose based in this glycopattern to form sialylated N-glycans or lewis antigen. To provide insights into the substitution and branching pattern of the monosaccharide constituents, the peaks which corresponding to the LacNAc-contained N-glycans observed in the MS spectrum were assigned to tandem MS analysis. The MS/MS spectra of the precursor ions *m/z*: 2594.910, 2976.037, 2996.102, and 3559.248 were illustrated in Figure S1.

### Verification of Aberrant Expression of LacNAc in GC Cells

**Figure 4:**
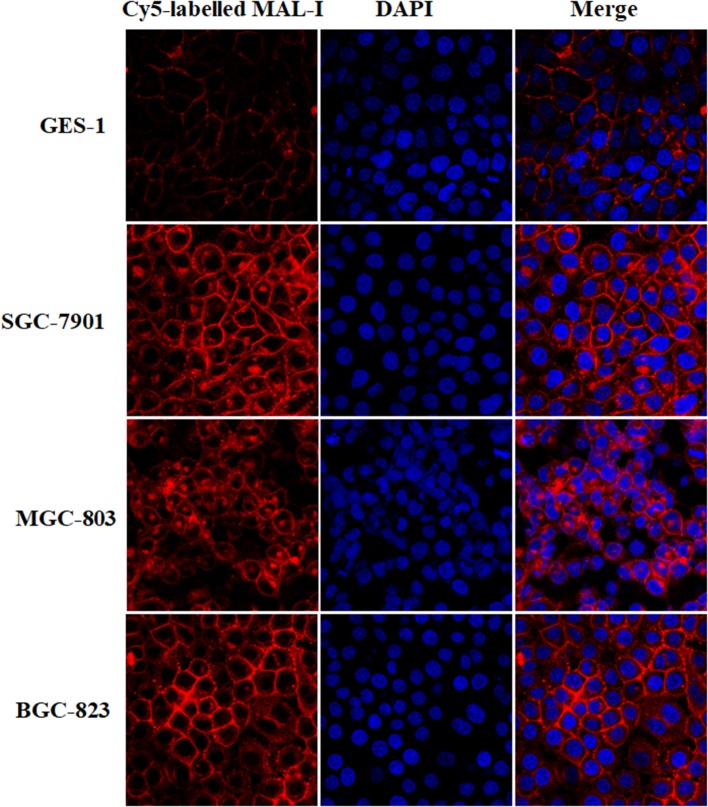
The glycopattern of LacNAc identified by MAL-I with abnormal accumulation in GC cells compared with normal control. The cells were fixed by 4% (*v/v*) paraformaldehyde solution and stained with Cy5-labeled MAL-I at a final concentration of 100 μg/mL. The images were acquired at the same exposure time and shown on the same scale for MAL-I with Cy5 channel, DAPI channel as well as Cy5 and DAPI merge channel, respectively.

According to the results of lectin microarrays, there was an abnormal accumulation of LacNAc (as identified by MAL-I) in tumor cells and tissue. Next, lectin cytochemistry was carried out to further investigate the glycan profiles in cellular level. Cy5-labeled MAL-I was used to assess the glycosylation patterns in GC and normal cells. This revealed that the staining intensities of MAL-I were significantly increased in GC cells compared to GES-1. In addition, lectin MAL-I showed abnormal accumulation in the cytomembrane and ECM in SGC-7901, MGC803, and BGC-823 compared to GES-1 (Figure 4). These results indicated that the presence of LacNAc showed an increase in GC cells compared to controls, which were consistent with results of lectin microarrays.

## Discussion

It is well-known that aberrant glycosylation not only represents characteristic of cancer, but also plays a vital role in key pathological steps of tumor progression. In the present study, we found that the relative abundance of T antigen (identified by ACA and PNA) as well as LacNAc (identified by MAL-I) showed significantly increased in both GC cells and tissue from patients at stage II and III compared to their corresponding controls. T antigen is one type of truncated O-glycan, which is considered to be associated with various types of cancer (31–33). Some studies reported that the high expressions of T antigen is highly associated with the metastatic and poor survival in prostate cancer and the interaction between galectin-4 and T antigen may contribute to this process (34, 35). Besides that, the interaction between T antigen and galectin-3 influences adhesion of tumor cells to the endothelium, which could promote metastasis (36). The Tn antigen (GalNAc-Ser/Thr) which is known as a tumor-associated carbohydrate antigen. The sialylated Tn antigen is expressed by more than 80% of human carcinomas and is linked to poor prognosis in cancer patients. Also the sialylated Tn antigen in serum could be a useful for detecting peritoneal metastases of GC (37, 38). Our results indicated that sialylated Tn antigen showed significantly increased in GC patients with advanced stage. Yamashita et al. reported that Tn/ sTn antigen which recognized by VVA was remarkably attenuated in the GC tumor cells of the metastatic lymph nodes (39). Knockdown of O-glycosyltransferase GALNT3 resulted in increasing the expression of poorly differentiated pancreatic ductal adenocarcinoma (PDAC) markers and influencing the O-glycans (Tn and T) expression in EGFR and Her2 in PDAC cells (40). Our results also revealed that the relative abundance of glycopatterns recognized by PHA-E+L show decreased in GC cells compared to normal control. Similar to our finding, the level of bisecting GlcNAc, bi-antennary N-glycans, tri- and tetra-antennary complex-type N-glycan which recognized by PHA-E+L showed decreased in GC cells (SGC-7901, HGC-27, and MGC-803) and tissue compared to GES-1 and gastric ulcer tissue (41, 42). It demonstrated that the addition of the bisecting GlcNAc to complex N-glycans of mammary tumor cell glycoprotein receptors could retard tumor progression by reducing growth factor signaling (43).

Our results revealed that more LacNAc contained N-glycans were characterized in tumor sample at stage III. Moreover, 10 neo-LacNAc-modified glycans emerged in tumor tissue at late stage, six of which were modified with sialic acids, or fucose based in this glycopattern to form sialylated N-glycans or lewis antigen. The phenomenon was commonly observed in various types of cancer cells (8, 44, 45). We concluded that the glycopattern of LacNAc serves as a scaffold for further modification such as sialic acid and tumor-associated carbohydrate antigen. Some studies revealed that the LacNAc could be modified with sialic acid to form NeuAcα2, 6Galβ1, 4GlcNAc, which is commonly overexpressed in gastrointestinal cancers correlating with poor prognosis, and this abnormal glycosylation could modulate invasiveness and stemness of colorectal cancer cells (46–49). In breast cancer, the ER-negative tumors are predicted to express more sLe^x^ antigen on lactosamine chains of N-/O-glycans than ER-positive tumors (50). Kunzke et al. reported that the high abundance of certain native glycans correlated with poor prognosis, distant metastases (pM) and expression of HER/2neu, EGFR and MIB1 in GC. It is important to notify the effect of LacNAc, since it is one type of Hex–HexAc and the high abundance of Hex–HexAc in tumor stroma regions was associated with poor prognosis of GC (51).

It known that neo-synthesis is the major mechanisms for generation of cancer-specific glycans, which is commonly observed in the advanced stages of cancer (8, 52, 53). During the development of cancer, certain alterations emerged in N-glycans such as the increase of branch structure and the mutation of the amount and the linkage of sialic acids (54–56). Some studies indicated that the overexpression of α2,3-linked terminal sialic acid epitopes causes a further invasive phenotype *in vitro* and *in vivo* in gastric cancer cells (47). The abundance of α2,3-linked sialic acids was proportional to the metastatic capacity of GC cells and the high expression of this glycopattern was highly correlated with lymph node metastasis. Elevating the expression of α2-3Sia in SGC-7901 cells will enhance cell migration and invasion ability (57). These neo-synthetic glycans are not only well-established signatures of malignant cell, but also affect cell recognition, cell–matrix interactions, and immune modulation. Eventually, this will lead to cancer progression and metastasis (58). Integrins are transmembrane receptors that bind extracellular matrix (ECM) components and trigger signaling cascades that regulate cellular events during development, normal homeostasis, and disease. The status of Glycosylation on integrins could influence the interactions between integrins and ECM proteins, especially, sialic acid could mask the underlying N-acetyllactosamine substrates and prevent the interaction between β1 subunit of integrins and Gal-3 (59). The interaction between galectin family and LacNAc has been reported to promote immune evasion of tumor cells. For instance, the poly N-acetyllactosamine, the polymers of LacNAc mediated binding of galectin-3 to MHC class I-related chain A (MICA) has been demonstrated to contribute to the survival of bladder tumor cells and prevent the NK-mediated killing of C2GnT-expressing bladder tumor cells (60, 61). Galectin-1 which expressed in cancer-associated fibroblasts (CAFs) could promote the invasiveness of GC cells by binding to LacNAc of β1 integrin on the cell surface (62, 63). The prostate cancer cells have been reported to resistant to NK cell toxicity and prolong the survival time of cancer cells (10).

In conclusion, we discovered that the presence of LacNAc is significantly increased in GC cells and tissue. It exhibits a coincidentally increasing trend accompanied with tumor progression. More LacNAc contained N-glycans were characterized in tumor sample with advance stage, 10 neo-LacNAc-contained N-glycans only can be detected in tumor tissue in the advanced stage of GC. Six of these neo-LacNAc-contained N-glycans are modified with sialic acid or fucose to form sialylated N-glycans or lewis antigen, which are commonly observed in various of cancer cells. We conclude that the aberrant expression of this glycopattern may serve as a scaffold for further modification such as sialic acid and tumor-associated carbohydrate antigen, and these neo-glycans may be involved in tumorigenesis and metastasis. Our findings provide useful information for us to understand the development of GC.

## Data Availability

The raw data supporting the conclusions of this manuscript will be made available by the authors, without undue reservation, to any qualified researcher.

## Ethics Statement

The collection of human tissues was carried out in accordance with the approved guidelines, approved by the Human Ethics Committee of all participating units (Northwest University, 1st People's Hospital of Chenzhou and Institute of Cancer Research, University of South China, Hunan), and informed consent was received from each participant.

## Author Contributions

HY and XLi designed the study. XLi and HX collected the tissue specimens and clinical information. HY, XLi, MC, FZ, XLiu, JY, YZ, JS, WC, HD, KZ, CZ, and JZ performed the experiments. All authors participated in literature research and data classification. HY, XLi, MC, and FZ wrote the manuscript. HX and ZL reviewed and edited the manuscript before submission.

### Conflict of Interest Statement

The authors declare that the research was conducted in the absence of any commercial or financial relationships that could be construed as a potential conflict of interest.

## Supplementary Material

Figure S1 MALDI-TOF/TOF-MS/MS analyzing of the N-glycan precursor ion from MS spectra. With example, the four N -glycan peaks **(A)** *m/z* 2594.910, **(B)** *m/z* 2976.037, **(C)** *m/z* 2996.102, and **(D)** *m/z* 3559.248, subjected to MS/MS analysis.

Table S1 The normalized fluorescence intensities from normal and GC cells by the lectin microarray analysis based on data of 37 lectins.

Table S2 Masses, compositions, and structures for the *N*-glycans found in normal and GC tissues. ND, no detected.
