# Supplementary figures and images for: Integrated Glycome Strategy for Characterization of Aberrant LacNAc Contained N-Glycans Associated With Gastric Carcinoma
Source: Front Oncol. 2019 Jul 10;9:636. doi: 10.3389/fonc.2019.00636 (PMC6636412; doi:10.3389/fonc.2019.00636)

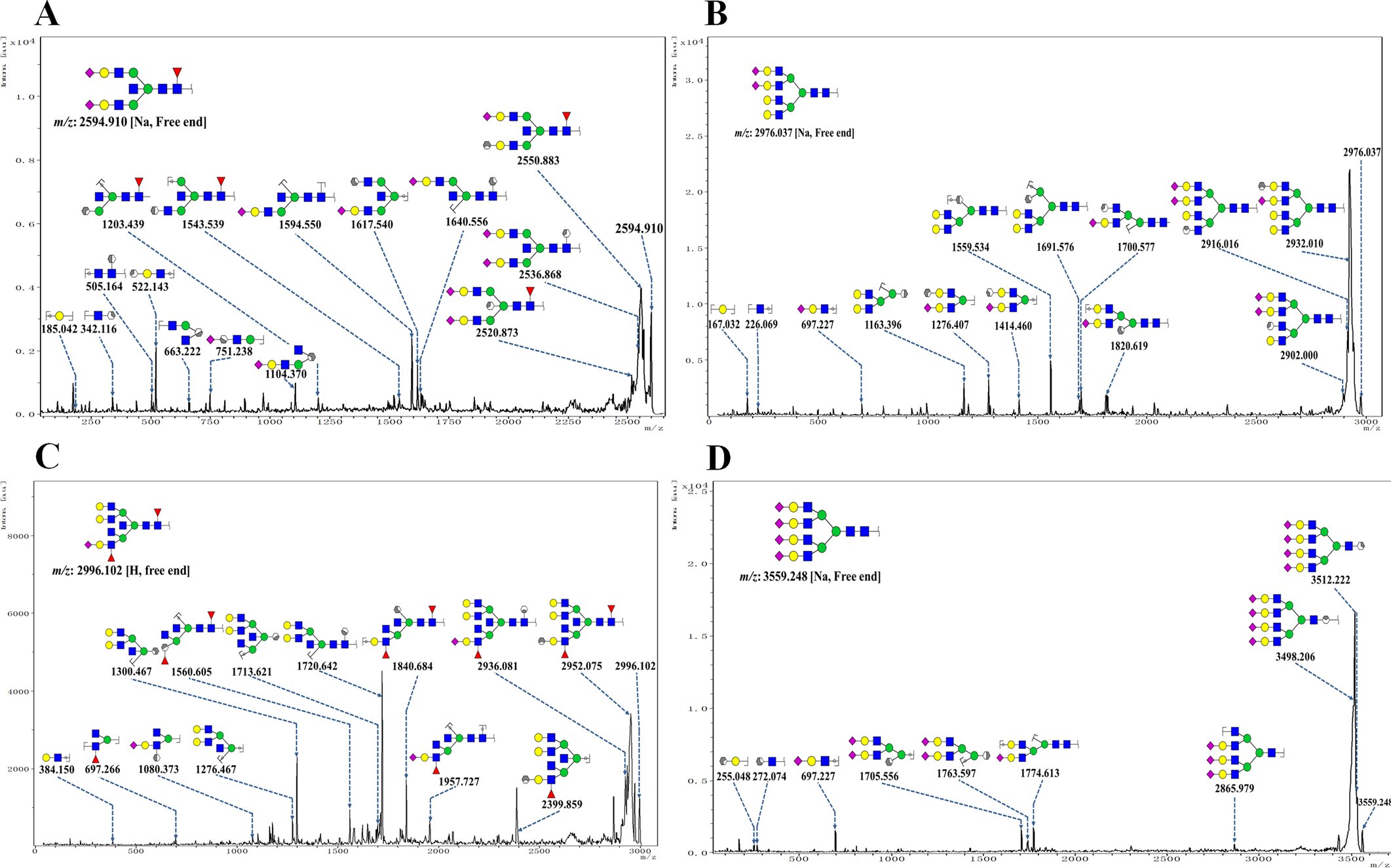

Supplement: Figure S1 — MALDI-TOF/TOF-MS/MS analyzing of the N-glycan precursor ion from MS spectra. With example, the four N -glycan peaks (A) m/z 2594.910, (B) m/z 2976.037, (C) m/z 2996.102, and (D) m/z 3559.248, subjected to MS/MS analysis. [file Image_1.JPEG]
