# Supplementary material for: Integrated Glycome Strategy for Characterization of Aberrant LacNAc Contained N-Glycans Associated With Gastric Carcinoma
Source: Front Oncol. 2019 Jul 10;9:636. doi: 10.3389/fonc.2019.00636 (PMC6636412; doi:10.3389/fonc.2019.00636)
Supplement: Table S1 — The normalized fluorescence intensities from normal and GC cells by the lectin microarray analysis based on data of 37 lectins. [file Table_1.DOCX]

**Table S1.** **The normalized fluorescence intensities from normal and GC cells by the lectin microarray analysis based on data of 37 lectins.**

| **Lectin** | **Specificity** | **GES-1** | **SGC-7901** | **MGC-803** | **BGC-823** |
| --- | --- | --- | --- | --- | --- |
| Jacalin | Galβ1-3GalNAcα-Ser/Thr(T), GalNAcα-Ser/Thr(Tn), GlcNAcβ1-3-GalNAcα-Ser/Thr(Core3), sialyl-T(ST). not bind to Core2, Core6, and sialyl-Tn (STn) | 0.029±0.001 | 0.031±0.001 | 0.031±0.000 | 0.031±0.003 |
| ECA | Galβ-1,4GlcNAc (type II), Galβ1-3GlcNAc (type I) | 0.034±0.001 | 0.027±0.005 | 0.026±0.005 | 0.026±0.001 |
| HHL | High-Mannose, Manα1-3Man, Manα1-6Man, Man5-GlcNAc2-Asn | 0.049±0.003 | 0.038±0.002 | 0.038±0.002 | 0.034±0.002 |
| WFA | Terminating in GalNAcα/β1-3/6Gal | 0.046±0.000 | 0.023±0.001 | 0.023±0.001 | 0.030±0.005 |
| GSL-II | GlcNAc and agalactosylated tri/tetra antennary glycans | 0.030±0.007 | 0.015±0.001 | 0.015±0.001 | 0.014±0.001 |
| MAL-II | Siaα2-3Galβ1-4Glc(NAc)/Glc, Siaα2-3Gal, Siaα2-3, Siaα2-3GalNAc | 0.032±0.005 | 0.024±0.002 | 0.024±0.002 | 0.022±0.001 |
| PHA-E | Bisecting GlcNAc, biantennary complex-type N-glycan | 0.042±0.005 | 0.019±0.001 | 0.019±0.001 | 0.021±0.001 |
| PTL-I | GalNAc, GalNAcα-1,3Gal, GalNAcα-1,3Galβ-1,3/4Glc | 0.025±0.001 | 0.021±0.000 | 0.021±0.000 | 0.020±0.000 |
| SJA | Terminal in GalNAc and Gal, anti-A and anti-B human blood group | 0.039±0.003 | 0.014±0.001 | 0.014±0.001 | 0.011±0.000 |
| PNA | Galβ1-3GalNAcα-Ser/Thr(T) | 0.020±0.001 | 0.017±0.002 | 0.017±0.002 | 0.015±0.002 |
| EEL | Galα1-3(Fucα1-2)Gal (blood group B antigen) | 0.044±0.001 | 0.039±0.003 | 0.038±0.003 | 0.041±0.003 |
| AAL | Fucα1-6 GlcNAc(core fucose), Fucα1-3(Galβ1-4)GlcNAc | 0.031±0.001 | 0.030±0.002 | 0.030±0.002 | 0.036±0.004 |
| LTL | Fucα1-3Galβ1-4GlcNAc, Fucα1-anti-H blood group specificity | 0.036±0.006 | 0.024±0.000 | 0.025±0.000 | 0.018±0.001 |
| MPL | Galβ1-3GalNAc, GalNAc | 0.030±0.003 | 0.025±0.002 | 0.025±0.003 | 0.021±0.011 |
| LEL | LacNAc and poly LacNAc, (GlcNAc)_2-4_ | 0.023±0.003 | 0.061±0.013 | 0.060±0.021 | 0.072±0.003 |
| GSL-I | αGalNAc, αGal, anti-A and B | 0.034±0.004 | 0.023±0.002 | 0.023±0.002 | 0.026±0.001 |
| DBA | αGalNAc, Tn antigen, GalNAcα1-3((Fucα1-2))Gal (blood group A antigen) | 0.032±0.003 | 0.032±0.002 | 0.032±0.001 | 0.037±0.006 |
| LCA | Fucα-1,6GlcNAc,α-D-Man, α-D-Glc | 0.014±0.003 | 0.015±0.001 | 0.015±0.002 | 0.017±0.001 |
| STL | trimers and tetramers of GlcNAc, core (GlcNAc) of N-glycan | 0.046±0.012 | 0.074±0.010 | 0.073±0.009 | 0.073±0.004 |
| PTL-II | Gal, blood group H , T-antigen | 0.017±0.001 | 0.021±0.001 | 0.021±0.002 | 0.019±0.001 |
| DSA | (GlcNAc) 2-4, polyLacNAc and LacNAc (NA3, NA4) | 0.027±0.004 | 0.029±0.001 | 0.030±0.002 | 0.028±0.001 |
| VVA | terminal GalNAc, GalNAcα-Ser/Thr(Tn), GalNAcα1-3Gal | 0.014±0.001 | 0.021±0.001 | 0.021±0.001 | 0.031±0.001 |
| MAL-I | Galβ-1,4GlcNAc | 0.015±0.001 | 0.029±0.002 | 0.029±0.002 | 0.028±0.002 |
| GNA | Manα1-3Man | 0.025±0.002 | 0.016±0.002 | 0.016±0.001 | 0.016±0.002 |
| NPA | High-Mannose, Manα1-6Man | 0.015±0.002 | 0.026±0.003 | 0.026±0.002 | 0.021±0.001 |
| ACA | Galβ1-3GalNAcα-Ser/Thr (T antigen), sialyl-T(ST) tissue staining patterns are markedly different than those obtained with either PNA or Jacalin | 0.020±0.000 | 0.051±0.002 | 0.061±0.002 | 0.053±0.001 |
| BPL | Galβ1-3GalNAc, Terminal GalNAc | 0.015±0.000 | 0.018±0.001 | 0.018±0.0001 | 0.012±0.001 |
| PHA-E+L | Bisecting GlcNAc, bi-antennary N-glycans, tri- and tetra-antennary complex-type N-glycan | 0.018±0.001 | 0.008±0.000 | 0.008±0.002 | 0.008±0.000 |
| SNA | Sia2-6Gal/GalNAc | 0.016±0.003 | 0.025±0.000 | 0.025±0.000 | 0.025±0.001 |
| RCA120 | β-Gal, Galβ-1,4GlcNAc (type II), Galβ1-3GlcNAc (type I) | 0.014±0.001 | 0.012±0.001 | 0.012±0.001 | 0.011±0.001 |
| BS-I | α-Gal, α-GalNAc, Galα-1,3Gal, Galα-1,6Glc | 0.028±0.003 | 0.040±0.001 | 0.039±0.001 | 0.034±0.002 |
| PSA | Fucα-1,6GlcNAc, α-D-Man, α-D-Glc | 0.018±0.002 | 0.017±0.000 | 0.017±0.001 | 0.012±0.000 |
| SBA | α- or β-linked terminal GalNAc, (GalNAc)n, GalNAcα1-3Gal, blood-group A | 0.026±0.001 | 0.018±0.001 | 0.018±0.001 | 0.015±0.001 |
| WGA | Multivalent Sia and (GlcNAc)_n_ | 0.027±0.002 | 0.031±0.003 | 0.031±0.002 | 0.024±0.003 |
| UEA-I | Fucα1-2Galβ1-4Glc(NAc) | 0.021±0.002 | 0.015±0.000 | 0.015±0.001 | 0.010±0.000 |
| PWM | (GlcNAc)_n_ and polyLacNAc | 0.028±0.001 | 0.038±0.001 | 0.038±0.001 | 0.029±0.002 |
| ConA | High-Mannose, Manα1-6(Manα1-3)Man, αMannose, αGlc | 0.020±0.000 | 0.034±0.001 | 0.024±0.000 | 0.057±0.004 |
