# Supplementary material for: Integrated Glycome Strategy for Characterization of Aberrant LacNAc Contained N-Glycans Associated With Gastric Carcinoma
Source: Front Oncol. 2019 Jul 10;9:636. doi: 10.3389/fonc.2019.00636 (PMC6636412; doi:10.3389/fonc.2019.00636)
Supplement: Table S2 — Masses, compositions, and structures for the N-glycans found in normal and GC tissues. ND, no detected. [file Table_2.DOCX]

**Table S2. Masses, compositions, and structures for the *N-*glycans found in normal and GC tissues.**

| No | Calculated m/z | Ion type | Predicted structure | Relative intensity | | | | | |
| --- | --- | --- | --- | --- | --- | --- | --- | --- | --- |
|  |  |  |  | **Stage I** | | **Stage II** | | **Stage III** | |
|  |  |  |  | **N(%)** | **I T(%)** | **N(%)** | **T(%)** | **N(%)** | **T(%)** |
| 1 | 1479.547 | [M+H]^+^ | 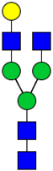 | 0.007 | 0.017 | ND | 0.009 | ND | ND |
| 2 | 1501.529 | [M+Na]^+^ |  | ND | ND | ND | ND | 0.022 | ND |
| 3 | 1608.589 | [M+H]^+^ | 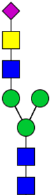 | 0.026 | ND | 0.012 | ND | 0.033 | 0.068 |
| 4 | 1622.555 | [M+Na]^+^ | 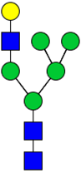 | ND | 0.065 | ND | ND | ND | ND |
| 5 | 1625.605 | [M+H]^+^ | 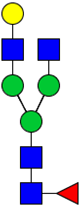 | ND | ND | ND | ND | ND | 0.015 |
| 6 | 1647.586 | [M+Na]^+^ |  | ND | ND | ND | ND | ND | 0.012 |
| 7 | 1641.599 | [M+H]^+^ | 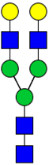 | 0.117 | 0.043 | ND | ND | 0.021 | 0.013 |
| 8 | 1666.631 | [M+H]^+^ | 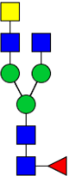 | ND | ND | ND | 0.009 | ND | ND |
| 9 | 1682.626 | [M+H]^+^ | 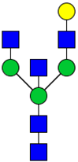 | ND | ND | ND | 0.098 | ND | ND |
| 10 | 1743.581 | [M+Na]^+^ | 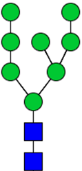 | ND | 0.064 | 0.015 | ND | ND | 0.022 |
| 11 | 1793.644 | [M+Na]^+^ | 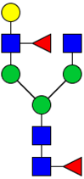 | 0.012 | ND | 0.065 | 0.055 | ND | ND |
| 12 | 1803.652 | [M+H]^+^ | 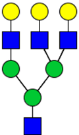 | 0.017 | 0.016 | 0.015 | ND | ND | 0.023 |
| 13 | 1825.634 | [M+Na]^+^ |  | ND | 0.023 | ND | ND | ND | ND |
| 14 | 1809.639 | [M+Na]^+^ | 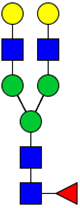 | ND | ND | ND | 0.026 | ND | ND |
| 15 | 1817.632 | [M+H]^+^ | 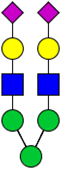 | ND | ND | ND | 0.025 | 0.034 | ND |
| 16 | 1850.666 | [M+Na]^+^ | 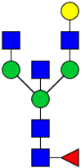 | 0.028 | 0.097 | ND | 0.081 | 0.069 | 0.024 |
| 17 | 1876.694 | [M+H]^+^ | 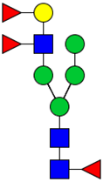 | ND | 0.025 | ND | ND | ND | ND |
| 18 | 1914.671 | [M+Na]^+^ | 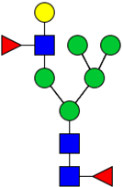 | ND | ND | ND | 0.011 | ND | 0.014 |
| 19 | 1930.623 | [M+Na]^+^ | 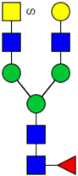 | ND | ND | ND | 0.012 | ND | ND |
| 20 | 1954.677 | [M+Na]^+^ | 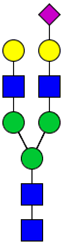 | ND | ND | ND | 0.031 | ND | ND |
| 21 | 1995.703 | [M+Na]^+^ | 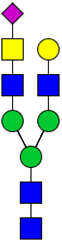 | 0.020 | ND | ND | ND | ND | ND |
| 22 | 2034.634 | [M+Na]^+^ | 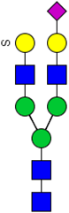 | ND | ND | ND | ND | ND | 0.068 |
| 23 | 2085.760 | [M+Na]^+^ | 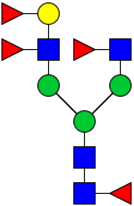 | 0.044 | ND | ND | ND | ND | ND |
| 24 | 2088.785 | [M+H]^+^ | 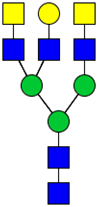 | ND | ND | 0.021 | 0.011 | 0.163 | 0.100 |
| 25 | 2100.735 | [M+Na]^+^ | 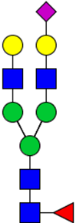 | ND | ND | 0.011 | ND | ND | ND |
| 26 | 2110.767 | [M+Na]^+^ | 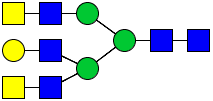 | 0.010 | ND | ND | 0.074 | 0.049 | ND |
| 27 | 2136.795 | [M+H]^+^ | 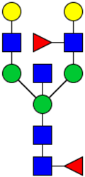 | 0.013 | ND | 0.014 | ND | ND | 0.020 |
| 28 | 2152.790 | [M+H]^+^ | 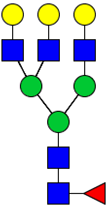 | ND | ND | ND | 0.012 | ND | ND |
| 29 | 2157.756 | [M+Na]^+^ | 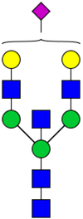 | ND | 0.015 | ND | ND | 0.020 | ND |
| 30 | 2198.783 | [M+Na]^+^ | 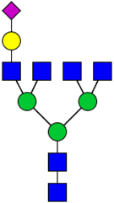 | ND | ND | ND | ND | 0.073 | 0.023 |
| 31 | 2204.746 | [M+Na]^+^ | 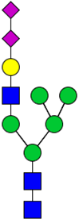 | ND | 0.109 | ND | ND | 0.019 | ND |
| 32 | 2223.790 | [M+H]^+^ | 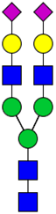 | ND | 0.064 | ND | 0.010 | ND | ND |
| 33 | 2245.772 | [M+Na]^+^ |  | 0.015 | ND | 0.029 | 0.013 | ND | ND |
| 34 | 2256.824 | [M+Na]^+^ | 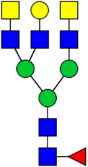 | 0.047 | 0.014 | 0.045 | ND | 0.020 | 0.017 |
| 35 | 2262.788 | [M+Na]^+^ | 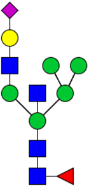 | ND | ND | 0.013 | ND | ND | ND |
| 36 | 2275.869 | [M+H]^+^ | 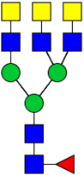 | ND | ND | ND | 0.022 | ND | ND |
| 37 | 2328.846 | [M+Na]^+^ | 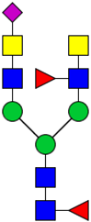 | ND | 0.015 | ND | ND | 0.025 | 0.029 |
| 38 | 2332.891 | [M+H]+ | 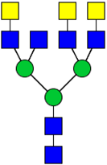 | ND | ND | ND | ND | ND | 0.017 |
| 39 | 2350.804 | [M+Na]^+^ | 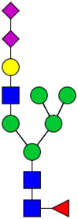 | ND | 0.022 | ND | ND | 0.028 | ND |
| 40 | 2380.901 | [M+H]^+^ | 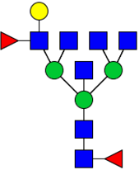 | ND | ND | ND | 0.010 | ND | ND |
| 41 | 2385.843 | [M+H]+ | 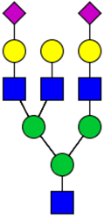 | 0.014 | ND | 0.150 | ND | ND | ND |
| 42 | 2407.825 | [M+Na]^+^ |  | ND | ND | 0.021 | ND | ND | ND |
| 43 | 2401.862 | [M+Na]^+^ | 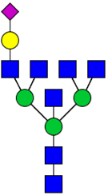 | 0.009 | 0.029 | ND | ND | ND | ND |
| 44 | 2440.835 | [M+Na]^+^ | 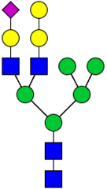 | ND | ND | ND | 0.012 | ND | ND |
| 45 | 2465.867 | [M+Na]^+^ | 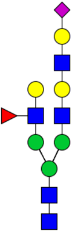 | ND | ND | 0.012 | ND | ND | ND |
| 46 | 2473.883 | [M+Na]^+^ | 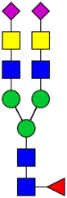 | ND | 0.012 | ND | ND | ND | 0.015 |
| 47 | 2478.949 | [M+H]+ | 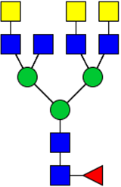 | ND | ND | 0.010 | ND | ND | ND |
| 48 | 2500.931 | [M+Na]^+^ |  | 0.017 | ND | 0.013 | ND | ND | ND |
| 49 | 2490.899 | [M+Na]^+^ | 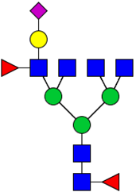 | ND | 0.024 | ND | ND | ND | 0.011 |
| 50 | 2546.876 | [M+H]+ | 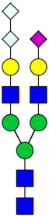 | ND | ND | ND | ND | ND | 0.11 |
| 51 | 2558.948 | [M+H]+ | 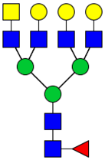 | ND | ND | ND | ND | 0.014 | 0.012 |
| 52 | 2567.985 | [M+H]+ | 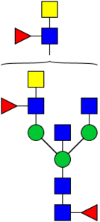 | ND | 0.024 | ND | ND | 0.035 | 0.014 |
| 53 | 2578.915 | [M+Na]^+^ | 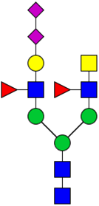 | ND | ND | ND | ND | ND | 0.015 |
| 54 | 2586.893 | [M+Na]^+^ | 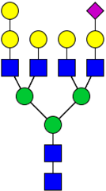 | 0.047 | ND | ND | ND | ND | ND |
| 55 | 2588.922 | [M+Na]+ | 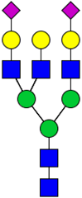 | ND | ND | ND | ND | ND | 0.012 |
| 56 | 2594.910 | [M+Na]^+^ | 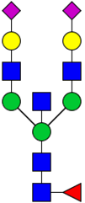 | ND | 0.035 | ND | ND | ND | ND |
| 57 | 2647.985 | [M+H]^+^ | 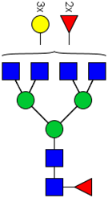 | ND | ND | ND | 0.015 | ND | ND |
| 58 | 2669.967 | [M+Na]^+^ | 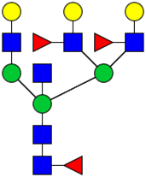 | ND | ND | ND | 0.007 | ND | ND |
| 59 | 2705.006 | [M+H]^+^ | 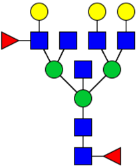 | 0.058 | ND | 0.051 | ND | ND | ND |
| 60 | 2762.028 | [M+H]+ | 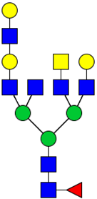 | ND | ND | ND | ND | ND | 0.022 |
| 61 | 2776.007 | [M+H]^+^ | 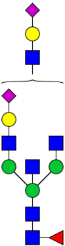 | ND | ND | ND | 0.024 | ND | ND |
| 62 | 2799.009 | [M+Na]^+^ |  | ND | ND | ND | 0.013 | ND | ND |
| 63 | 2819.049 | [M+H]^+^ | 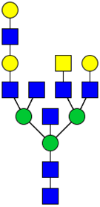 | ND | ND | ND | ND | 0.014 | 0.016 |
| 64 | 2830.999 | [M+Na]^+^ | 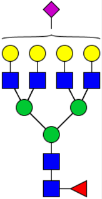 | 0.015 | ND | 0.020 | ND | ND | ND |
| 65 | 2846.994 | [M+Na]^+^ | 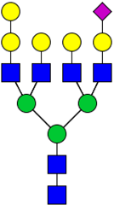 | ND | ND | 0.039 | 0.066 | 0.012 | 0.032 |
| 66 | 2866.039 | [M+H]^+^ | 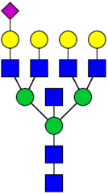 | 0.009 | 0.012 | ND | 0.039 | 0.027 | ND |
| 67 | 2873.046 | [M+Na]^+^ | 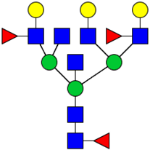 | ND | ND | ND | ND | ND | 0.014 |
| 68 | 2929.047 | [M+Na]^+^ | 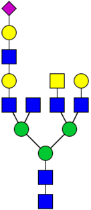 | ND | ND | ND | ND | 0.013 | 0.009 |
| 69 | 2960.042 | [M+Na]^+^ | 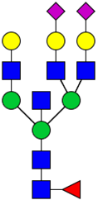 | 0.007 | ND | ND | ND | ND | 0.016 |
| 70 | 2976.037 | [M+Na]^+^ | 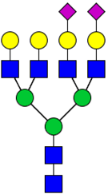 | 0.059 | 0.038 | ND | ND | 0.041 | 0.078 |
| 71 | 2993.052 | [M+Na]^+^ | 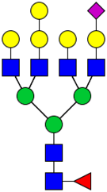 | ND | ND | ND | ND | ND | 0.023 |
| 72 | 2996.102 | [M+H]^+^ | 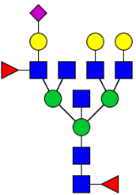 | 0.017 | 0.035 | ND | ND | 0.068 | 0.011 |
| 73 | 3042.071 | [M+H]^+^ | 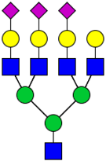 | ND | 0.011 | ND | ND | 0.009 | ND |
| 74 | 3321.228 | [M+H]^+^ | 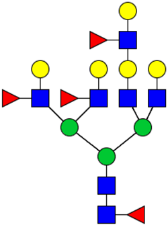 | ND | 0.011 | 0.011 | ND | ND | ND |
| 75 | 3430.205 | [M+Na]^+^ | 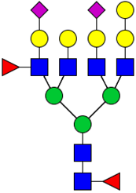 | 0.014 | ND | 0.015 | 0.026 | ND | ND |
| 76 | 3446.200 | [M+Na]^+^ | 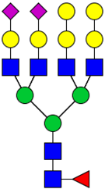 | ND | ND | ND | 0.009 | ND | ND |
| 77 | 3455.237 | [M+Na]^+^ | 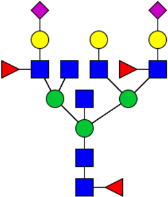 | 0.005 | ND | 0.004 | ND | ND | ND |
| 78 | 3559.248 | [M+Na]^+^ | 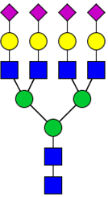 | ND | 0.012 | ND | ND | 0.009 | 0.004 |

ND: no detected.
